# Supplementary material for: Comparative multiomics analysis of cell physiological state after culture in a basket bioreactor
Source: Sci Rep. 2022 Nov 23;12:20161. doi: 10.1038/s41598-022-24687-4 (PMC9686226; doi:10.1038/s41598-022-24687-4)
Supplement: Supplementary file 1 — Supplementary Information 1. [file 41598_2022_24687_MOESM1_ESM.zip › raw data/Transcriptomic raw data/6.Enrichment/1.GO/TE1vsTC1/GSEA/TE1/CELLULAR_PROTEIN_LOCALIZATION/CELLULAR_PROTEIN_LOCALIZATION(GO_0034613).html]

Details for gene set CELLULAR\_PROTEIN\_LOCALIZATION(GO:0034613)[GSEA]


|  || Dataset | readcount.sample |
| Phenotype | sample.cls |
| Upregulated in class | TE1 |
| GeneSet | CELLULAR\_PROTEIN\_LOCALIZATION(GO:0034613) |
| Enrichment Score (ES) | 0.3583252 |
| Normalized Enrichment Score (NES) | 1.8025233 |
| Nominal p-value | 0.0 |
| FDR q-value | 0.21522455 |
| FWER p-Value | 0.494 |
Table: GSEA Results Summary

  

Fig 1: Enrichment plot: CELLULAR\_PROTEIN\_LOCALIZATION(GO:0034613)
  
Profile of the Running ES Score & Positions of GeneSet Members on the Rank Ordered List

  

| PROBE | DESCRIPTION   (from dataset) | GENE SYMBOL | GENE\_TITLE | RANK IN GENE LIST | RANK METRIC SCORE | RUNNING ES | CORE ENRICHMENT || 1 | 103222840 | LOC103222840 |  |  | 441 | Infinity | -0.0197 | Yes |
| 2 | 103225893 | LOC103225893 |  |  | 560 | Infinity | -0.0249 | Yes |
| 3 | 103224720 | LOC103224720 |  |  | 680 | 7.298 | 0.0214 | Yes |
| 4 | 103231257 | LOC103231257 |  |  | 1095 | 3.305 | 0.0263 | Yes |
| 5 | 103240797 | LOC103240797 |  |  | 1560 | 2.400 | 0.0225 | Yes |
| 6 | 103236180 | LOC103236180 |  |  | 1731 | 2.265 | 0.0309 | Yes |
| 7 | 103232300 | LOC103232300 |  |  | 1824 | 2.207 | 0.0424 | Yes |
| 8 | 103228869 | SEC23A |  |  | 1967 | 2.101 | 0.0509 | Yes |
| 9 | 103240505 | IPO7 |  |  | 2289 | 1.938 | 0.0502 | Yes |
| 10 | 103229165 | AKAP5 |  |  | 2488 | 1.860 | 0.0545 | Yes |
| 11 | 103239820 | COPB1 |  |  | 2922 | 1.727 | 0.0473 | Yes |
| 12 | 103235715 | SRP72 |  |  | 3021 | 1.701 | 0.0550 | Yes |
| 13 | 103226057 | SEC61G |  |  | 3120 | 1.682 | 0.0625 | Yes |
| 14 | 103246817 | KDELR2 |  |  | 3306 | 1.644 | 0.0659 | Yes |
| 15 | 103247446 | LOC103247446 |  |  | 3369 | 1.633 | 0.0747 | Yes |
| 16 | 103240726 | AKAP12 |  |  | 3572 | 1.596 | 0.0769 | Yes |
| 17 | 119619579 | SRP9 |  |  | 3576 | 1.596 | 0.0881 | Yes |
| 18 | 103230793 | TOMM20 |  |  | 3669 | 1.580 | 0.0951 | Yes |
| 19 | 103220190 | XPO1 |  |  | 3715 | 1.571 | 0.1043 | Yes |
| 20 | 103242823 | CLTC |  |  | 3941 | 1.535 | 0.1050 | Yes |
| 21 | 103237959 | STAM |  |  | 4075 | 1.517 | 0.1098 | Yes |
| 22 | 103240502 | KPNA5 |  |  | 4156 | 1.508 | 0.1169 | Yes |
| 23 | 103244343 | SRP19 |  |  | 4184 | 1.505 | 0.1264 | Yes |
| 24 | 103218923 | LOC103218923 |  |  | 4287 | 1.487 | 0.1323 | Yes |
| 25 | 103241428 | SSR3 |  |  | 4441 | 1.468 | 0.1359 | Yes |
| 26 | 103228813 | SRP54 |  |  | 4484 | 1.462 | 0.1443 | Yes |
| 27 | 103235816 | USO1 |  |  | 4535 | 1.455 | 0.1524 | Yes |
| 28 | 103223321 | TOMM22 |  |  | 4770 | 1.426 | 0.1520 | Yes |
| 29 | 103245632 | AP4E1 |  |  | 4815 | 1.421 | 0.1601 | Yes |
| 30 | 103234478 | LOC103234478 |  |  | 4938 | 1.408 | 0.1646 | Yes |
| 31 | 103217575 | PGAP1 |  |  | 4994 | 1.402 | 0.1721 | Yes |
| 32 | 103219194 | CLTA |  |  | 5081 | 1.392 | 0.1781 | Yes |
| 33 | 103228184 | KPNA1 |  |  | 5210 | 1.378 | 0.1821 | Yes |
| 34 | 103231289 | SELENOS |  |  | 5220 | 1.377 | 0.1915 | Yes |
| 35 | 103244556 | SEC24A |  |  | 5284 | 1.370 | 0.1983 | Yes |
| 36 | 103242897 | TOM1L1 |  |  | 5505 | 1.349 | 0.1980 | Yes |
| 37 | 103227635 | DAG1 |  |  | 5709 | 1.330 | 0.1983 | Yes |
| 38 | 103215432 | XPO7 |  |  | 6061 | 1.296 | 0.1918 | Yes |
| 39 | 103222151 | IPO11 |  |  | 6147 | 1.289 | 0.1971 | Yes |
| 40 | 103214430 | KPNA3 |  |  | 6252 | 1.278 | 0.2015 | Yes |
| 41 | 103222967 | TNPO1 |  |  | 6265 | 1.276 | 0.2100 | Yes |
| 42 | 103225204 | KPNA6 |  |  | 6560 | 1.257 | 0.2057 | Yes |
| 43 | 103233854 | NXF1 |  |  | 6631 | 1.249 | 0.2114 | Yes |
| 44 | 103226389 | TOMM7 |  |  | 6643 | 1.248 | 0.2197 | Yes |
| 45 | 103228713 | IPO4 |  |  | 6683 | 1.244 | 0.2268 | Yes |
| 46 | 103230327 | IPO9 |  |  | 6820 | 1.235 | 0.2295 | Yes |
| 47 | 103245852 | SRP14 |  |  | 6858 | 1.232 | 0.2365 | Yes |
| 48 | 103214377 | COG3 |  |  | 6864 | 1.232 | 0.2450 | Yes |
| 49 | 103234972 | KDELR1 |  |  | 6939 | 1.226 | 0.2504 | Yes |
| 50 | 103236101 | SEC24B |  |  | 6965 | 1.224 | 0.2580 | Yes |
| 51 | 103232989 | BCAP31 |  |  | 6969 | 1.224 | 0.2665 | Yes |
| 52 | 103223509 | AP3B1 |  |  | 7008 | 1.220 | 0.2734 | Yes |
| 53 | 103235773 | LOC103235773 |  |  | 7075 | 1.215 | 0.2791 | Yes |
| 54 | 103223316 | KDELR3 |  |  | 7232 | 1.202 | 0.2806 | Yes |
| 55 | 103244420 | SNX2 |  |  | 7239 | 1.201 | 0.2888 | Yes |
| 56 | 103226195 | VPS41 |  |  | 7388 | 1.191 | 0.2906 | Yes |
| 57 | 103235185 | AP2A2 |  |  | 7512 | 1.180 | 0.2935 | Yes |
| 58 | 103230911 | CNST |  |  | 7744 | 1.162 | 0.2913 | Yes |
| 59 | 103214961 | SEC23B |  |  | 7812 | 1.156 | 0.2965 | Yes |
| 60 | 103220202 | PEX13 |  |  | 7877 | 1.153 | 0.3018 | Yes |
| 61 | 103244616 | CSE1L |  |  | 7905 | 1.150 | 0.3088 | Yes |
| 62 | 103243153 | KPNA2 |  |  | 8015 | 1.142 | 0.3120 | Yes |
| 63 | 103242051 | COPB2 |  |  | 8037 | 1.140 | 0.3191 | Yes |
| 64 | 103226516 | SEM1 |  |  | 8238 | 1.131 | 0.3181 | Yes |
| 65 | 103223741 | MTX3 |  |  | 8565 | 1.113 | 0.3114 | Yes |
| 66 | 103241393 | KPNA4 |  |  | 8569 | 1.113 | 0.3191 | Yes |
| 67 | 103241183 | TGFBRAP1 |  |  | 8943 | 1.089 | 0.3101 | Yes |
| 68 | 103233646 | AP3D1 |  |  | 9032 | 1.084 | 0.3139 | Yes |
| 69 | 103243578 | KPNB1 |  |  | 9047 | 1.083 | 0.3209 | Yes |
| 70 | 103226799 | LOC103226799 |  |  | 9050 | 1.082 | 0.3285 | Yes |
| 71 | 103236172 | SEC24D |  |  | 9057 | 1.082 | 0.3359 | Yes |
| 72 | 103233178 | AP1G1 |  |  | 9161 | 1.075 | 0.3389 | Yes |
| 73 | 103226904 | COPG2 |  |  | 9381 | 1.061 | 0.3366 | Yes |
| 74 | 103245499 | SNX1 |  |  | 9422 | 1.059 | 0.3423 | Yes |
| 75 | 103226712 | BCAP29 |  |  | 9554 | 1.051 | 0.3438 | Yes |
| 76 | 103248787 | SRPRA |  |  | 9555 | 1.051 | 0.3513 | Yes |
| 77 | 103230424 | TPR |  |  | 9717 | 1.040 | 0.3514 | Yes |
| 78 | 103218814 | IPO8 |  |  | 9799 | 1.035 | 0.3551 | Yes |
| 79 | 103223722 | COPA |  |  | 9930 | 1.026 | 0.3566 | Yes |
| 80 | 103243015 | SRP68 |  |  | 10052 | 1.016 | 0.3583 | Yes |
| 81 | 103242441 | LOC103242441 |  |  | 10225 | 1.006 | 0.3577 | No |
| 82 | 103230213 | XPO6 |  |  | 10631 | 0.982 | 0.3465 | No |
| 83 | 103242741 | AP2B1 |  |  | 11029 | 0.955 | 0.3355 | No |
| 84 | 103223285 | GGA1 |  |  | 11307 | 0.937 | 0.3297 | No |
| 85 | 103215992 | SEC24C |  |  | 11429 | 0.930 | 0.3308 | No |
| 86 | 103247047 | VPS16 |  |  | 11557 | 0.922 | 0.3317 | No |
| 87 | 103216529 | VTI1A |  |  | 11614 | 0.918 | 0.3357 | No |
| 88 | 103229212 | VTI1B |  |  | 11804 | 0.906 | 0.3336 | No |
| 89 | 103245518 | TLN2 |  |  | 11847 | 0.903 | 0.3381 | No |
| 90 | 103231443 | OXA1L |  |  | 12024 | 0.893 | 0.3366 | No |
| 91 | 103243049 | GGA3 |  |  | 12092 | 0.889 | 0.3398 | No |
| 92 | 103233984 | TNPO2 |  |  | 12213 | 0.881 | 0.3407 | No |
| 93 | 103219208 | TLN1 |  |  | 12323 | 0.874 | 0.3420 | No |
| 94 | 103231482 | AP1G2 |  |  | 12432 | 0.868 | 0.3433 | No |
| 95 | 103224962 | IPO13 |  |  | 12523 | 0.861 | 0.3454 | No |
| 96 | 103239946 | LOC103239946 |  |  | 12979 | 0.834 | 0.3309 | No |
| 97 | 103228660 | COPG1 |  |  | 13010 | 0.832 | 0.3354 | No |
| 98 | 103223009 | CLTCL1 |  |  | 13356 | 0.810 | 0.3257 | No |
| 99 | 103225827 | AGRN |  |  | 13623 | 0.794 | 0.3194 | No |
| 100 | 103218163 | RAMP1 |  |  | 14052 | 0.765 | 0.3056 | No |
| 101 | 103223888 | MTX1 |  |  | 14343 | 0.750 | 0.2979 | No |
| 102 | 103223229 | TOM1 |  |  | 14425 | 0.744 | 0.2995 | No |
| 103 | 103229082 | TOMM20L |  |  | 14569 | 0.735 | 0.2983 | No |
| 104 | 103243727 | HGS |  |  | 15045 | 0.705 | 0.2820 | No |
| 105 | 103248617 | VPS11 |  |  | 15255 | 0.692 | 0.2775 | No |
| 106 | 103223137 | AP1B1 |  |  | 15531 | 0.673 | 0.2699 | No |
| 107 | 103243189 | LOC103243189 |  |  | 15880 | 0.648 | 0.2589 | No |
| 108 | 103231094 | AP3B2 |  |  | 16105 | 0.635 | 0.2533 | No |
| 109 | 103235543 | AP2A1 |  |  | 16512 | 0.603 | 0.2394 | No |
| 110 | 103224181 | AP4B1 |  |  | 16688 | 0.593 | 0.2357 | No |
| 111 | 103245809 | VPS18 |  |  | 16953 | 0.573 | 0.2280 | No |
| 112 | 103232389 | LOC103232389 |  |  | 17277 | 0.551 | 0.2174 | No |
| 113 | 103245028 | CLTB |  |  | 17433 | 0.539 | 0.2142 | No |
| 114 | 103227179 | LOC103227179 |  |  | 18380 | 0.452 | 0.1750 | No |
| 115 | 103230141 | LOC103230141 |  |  | 18982 | 0.392 | 0.1508 | No |
| 116 | 103232396 | NXF3 |  |  | 20526 | 0.185 | 0.0829 | No |
| 117 | 103244795 | CD74 |  |  | 20593 | 0.173 | 0.0811 | No |
| 118 | 103236685 | LOC103236685 |  |  | 21186 | 0.000 | 0.0546 | No |
Table: GSEA details
[plain text format]

  

Fig 2: CELLULAR\_PROTEIN\_LOCALIZATION(GO:0034613)
  
Blue-Pink O' Gram in the Space of the Analyzed GeneSet

  

Fig 3: CELLULAR\_PROTEIN\_LOCALIZATION(GO:0034613): Random ES distribution
  
Gene set null distribution of ES for
 **CELLULAR\_PROTEIN\_LOCALIZATION(GO:0034613)**
